# β-TCP/HA with or without enamel matrix proteins for maxillary sinus floor augmentation: a histomorphometric analysis of human biopsies

**DOI:** 10.1186/s40729-017-0080-8

**Published:** 2017-05-04

**Authors:** James Carlos Nery, Luís Antônio Violin Dias Pereira, George Furtado Guimarães, Cassio Rocha Scardueli, Fabiana Mantovani Gomes França, Rubens Spin-Neto, Andreas Stavropoulos

**Affiliations:** 1 Department of Implantology, São Leopoldo Mandic Research Center, Brasília, DF Brazil; 2 Department of Biochemistry and Tissue Biology, UNICAMP – State University of Campinas, Institute of Biology, Campinas, São Paulo Brazil; 3 Department of Periodontology, UNESP – Univ. Estadual Paulista, Araraquara Dental School, Araraquara, São Paulo Brazil; 4 Department of Dentistry and Oral Health – Oral Radiology, Aarhus University, Aarhus, Denmark; 5 Department Periodontology – Faculty of Odontology, Malmö University, Malmö, Sweden; 6 Implant Center, SEPS 710/910, Lotes CD, Office 226, CEP: 70390-108 Brasília, DF Brazil

**Keywords:** Bone substitute, Maxillary sinus floor, Enamel matrix proteins, Histomorphometry, Human

## Abstract

**Background:**

It is still unclear whether enamel matrix proteins (EMD) as adjunct to bone grafting enhance bone healing. This study compared histomorphometrically maxillary sinus floor augmentation (MSFA) with β-TCP/HA in combination with or without EMD in humans.

**Methods:**

In ten systemically healthy patients needing bilateral MSFA, one side was randomly treated using β-TCP/HA mixed with EMD (BC + EMD) and the other side using only β-TCP/HA (BC). After 6 months, biopsies were harvested from grafted areas during implant installation, being histologically and histomorphometrically analyzed. Differences between the groups considering new bone formation, soft tissues, and remaining BC were statistically evaluated.

**Results:**

All patients showed uneventful healing after MSFA, and dental implant installation was possible in all patients after 6 months. Histological analysis showed newly formed bone that was primarily woven in nature; it was organized in thin trabeculae, and it was occasionally in contact with residual bone substitute particles, which appeared in various forms and sizes and in advanced stage of degradation. Mean bone area was 43.4% (CI95 38.9; 47.8) for the BC group and 43.0% (CI95 36.6; 49.5) for the BC + EMD group. Mean soft tissue area was 21.3% (CI95 16.5; 26.2) for BC group and 21.5% (CI95 17.7; 25.3) for BC + EMD group, while the remaining biomaterial was 35.3% (CI95 36.6; 49.5) and 35.5% (CI95 29.6; 41.3) for BC and BC + EMD group, respectively.

**Conclusions:**

MSFA with BC resulted in adequate amounts of new bone formation allowing successful implant installation; adding EMD did not have a significant effect.

## Background

Reconstruction of the edentulous and severely atrophied posterior maxilla is often performed by means of maxillary sinus floor augmentation in combination with dental implants [1, 2]. Various bone graft materials are typically used for enhancing bone formation within the sinus cavity; autogenous bone (AB) is considered as the gold standard due to its osteogenic, osteoinductive, and osteoconductive properties [3–5]. However, harvesting AB from intraoral sites is associated with a number of pitfalls such as donor site morbidity, surgical complications, and extra time, while in some occasions there is limited availability in intraoral bone [6]. Furthermore, the available scientific evidence neither supports nor refutes the superiority of AB over other graft materials for maxillary sinus augmentation with regard to implant survival or complications at the recipient site [7].

Various bone substitute materials, that attempt to incorporate several features of AB, have been evaluated with the aim to replace AB grafting [1]. Biphasic calcium phosphate has been widely used as a bone substitute in orthopedics, periodontology, and maxillofacial and oral surgery. It has been shown to be a safe biocompatible scaffold supporting new bone formation, used either alone or in combination with growth factors [8, 9]. Bone Ceramic® (BC; Straumann, Basel, Switzerland) is among the biphasic calcium phosphates currently available in the market. It is a fully synthetic bone graft substitute of medical grade purity in particulate form (particle size 500–1000 μm), consisting of 60% hydroxyapatite (HA) and 40% beta tri-calcium phosphate. Studies have shown that BC acts as osteoconductive material when used for maxillary sinus floor augmentation [4, 10].

An enamel matrix protein derivative (EMD; Emdogain, Straumann, Basel, Switzerland) has been used in periodontal regenerative procedures for over 20 years, and it has been shown to efficiently enhance the outcome of healing [11, 12]. Although the few available preclinical studies have not shown any clear benefit when EMD was used for bone regeneration, emerging evidence shows that EMD upregulates the expression of several chemokines and growth factors relevant for bone wound healing [13]. In this context, clinical testing on the possible potential of EMD to enhance bone formation in other types of bone defects (i.e., non-periodontal) is sparse and the results are unclear [14].

The aim of the present study was to compare histomorphometrically the outcome of maxillary sinus floor augmentation with β-TCP/HA with or without enamel matrix proteins (BC + EMD and EMD, respectively) in humans.

## Methods

This research project was approved by the Ethics Committee of the School of Dentistry and Dental Research Center São Leopoldo Mandic, Brazil, under the protocol 2010/0360.

### Sample definition

Ten consecutive patients (age range 35–75 years) with the need of bilateral maxillary sinus floor augmentation prior to the placement of four dental implants (two in each side of posterior maxilla) were selected for the study. The main inclusion criterion was a vertical dimension of the residual alveolar bone between 3 and 5 mm in the sites selected for implant placement in the posterior maxilla, as assessed on a cone beam CT. Only patients with no need for additional bone augmentation (i.e., lateral or vertical) were included. The patients did not suffer from any systemic disease that might interfere with bone healing (e.g., uncontrollable diabetes; osteoporosis) and did not smoke more than 10 cigarettes per day. Sample size calculation was based on the statistical mean and standard deviation of percent new bone formation within the augmented maxillary sinus, reported previously in a similar study including histomorphometric evaluation [15].

### Maxillary sinus floor augmentation, biopsy harvesting, and dental implant placement

All patients received systemic antibiotics (amoxicillin 500 mg, every 8 h for 7 days) and anti-inflammatory drugs (nimesulide 100 mg twice daily for 5 days), starting all the medication 1 h before surgery. Patients were also prescribed analgesics (paracetamol 750 mg, max. four times a day) if there was pain. Chlorexidine digluconate 0.12% mouth rinses, four times daily, were also prescribed for 14 days post-operatively.

Surgery was planned using cone beam CT images (i-CAT, Image Sciences International, USA) with 0.25 mm voxel size, in 1-mm-thick sections, generated every 1 mm in the region of interest (posterior maxilla). After extra and intraoral disinfection of the operating field, local anesthesia was administered using lidocaine hydrochloride 2% with epinephrine 1:100.000 (DFL Industry and Trade, Rio de Janeiro, Brazil). Maxillary sinus floor augmentation with a lateral window technique was performed, and each of the sinuses received either β-TCP/HA (Straumann® BoneCeramic, Basel, Switzerland – BC group) or β-TCP/HA manually mixed using a periosteal elevator with EMD (Straumann® Emdogain, Basel, Switzerland), in a proportion of 1 g of BC for 0.3 ml of EMD (BC + EMD group), in a random fashion (by tossing a coin) and using a split-mouth design. In both groups, a very limited amount of sterile ;physiological saline solution (NaCl 0.9%) was added to the graft material mixture, insufficient amount to provide the consistency needed to ease handling and transferring into the sinus. No membrane or other material was used for closing the lateral window. After flap repositioning, closure was performed using simple interrupted nylon sutures (4-0, Ethicon, Johnson & Johnson). No radiographic examination immediately after sinus augmentation procedure was undertaken.

Six months after grafting, another CBCT examination was carried out for implant planning. In the sequence, following the previously described antiseptic and anesthetic procedures, two implants with a sand-blasted and acid etching surface were installed in each of the grafted sinuses, i.e., 40 implants in total (32—Neoporous, Neodent, Curitiba, Paraná, Brazil; 8—SLA, Straumann, Basel, Switzerland). A 10-mm-long cylindrical bone biopsy was harvested using a 2-mm internal diameter trephine bur during preparation for the most anterior implant site (i.e., two biopsies were retrieved from each patient). Six months later, the prosthetic rehabilitation of the patient was performed.

### Biopsy handling and evaluation

Immediately after retrieval, the apical aspect of the harvested biopsies was marked using India ink, to be used as a guide during histological evaluation. The biopsies were routinely processed (maintained in formaldehyde during 2 days, washed, and decalcified using EDTA solution, under continuous shaking, for 2 months) and embedded in paraffin. Six 6-μm-thick sections representing the central aspect of the cylindrical biopsy were obtained from each biopsy. These sections were stained using hematoxylin-eosin and were used for histological and histomorphometric analyses. Images were acquired using a DIASTAR light microscope (Leica Reichert & Jung products, Germany) connected to a Leica Microsystems DFC-300-FX digital camera (Leica Microsystems, Germany). Additional sections were stained using picrosirius-hematoxylin for microscopic examination under polarized light.

**Fig. 1:**
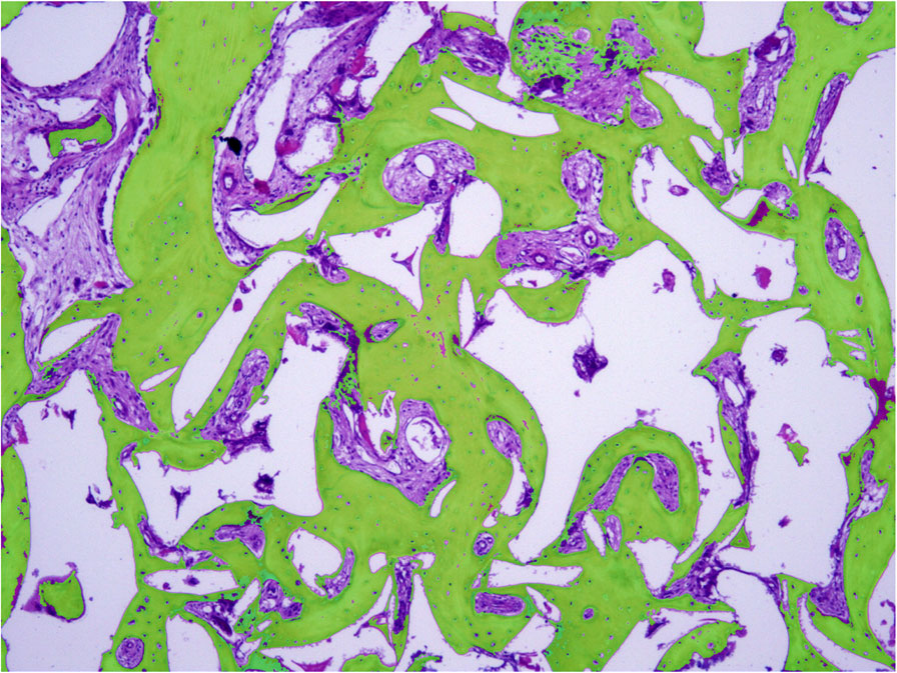
Histomicrograph illustrating the various tissue areas measured on the sections: newly formed bone (*green mask*), soft tissues (*purple mask*), and “others”, including residual bone substitute particles and empty spaces either due to removal of the bone substitute particles during to the decalcification processing or due to artifacts (*white mask*)

From the entire biopsy, only the 6 mm towards the apical aspect was considered as the region of interest (ROI), in order to allow visualization of approximately 80% of grafted bone and 20% of resident bone. Histological evaluation assessed morphological characteristics of the newly formed bone, remaining grafted material, integration of the grafted material with the newly formed bone, soft tissues, and local inflammation. Also, the newly formed bone was assessed regarding the aggregation and organization of the collagen bundles, reflected in the variation in birefringence intensity. The relative amounts (%) of bone, soft tissues, and “other material” (i.e., remaining grafting material or empty spaces due removal of the grafting material during histological processing, artifacts, and debris), within the ROI were planimetrically estimated using ImageJ (NIH, Bethesda, MD, USA) (Fig. 1).

### Data analysis

The data for each tissue component from the three histological sections were averaged to represent the biopsy. Commercially available software (GraphPad Prism 5.0 for Windows, GraphPad Software Inc., USA) was utilized for statistical comparisons between groups and for drawing the graphics. The assumption of normality was checked using D’Agostino & Pearson omnibus test. The data for each evaluated tissue, for BC and BC + EMD groups were analyzed as two paired samples from normal distributions based on a paired *t* test. Estimates were given with 95% confidence intervals, and statistical significance was set at 5% (*p* < 0.05).

## Results

### Clinical evaluation

All ten patients showed uneventful healing after the sinus floor augmentation procedure as well as after dental implant placement, with no overt postoperative inflammation or infection. Consistently, in all ten patients, no significant jiggling of the drill was noticed during biopsy harvesting, while subjective drilling resistance during implant placement was similar in both groups and all implants had appropriate primary stability as judged clinically. Further, even though bone substitute particles could still be recognized in the retrieved biopsy, all particles appeared well integrated in the biopsy tissue.

### Histological evaluation

**Fig. 2:**
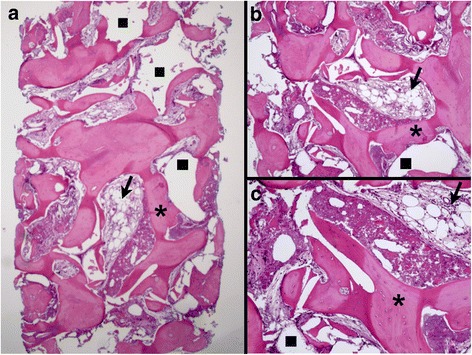
Histomicrograph of a biopsy from the BC group. **a** Overview—×25 magnification; **b** ×30 magnification; **c** ×60 magnification. Areas corresponding to BC removed during histological processing (*square*) in direct contact with newly formed bone (*asterisk*), containing a large number of osteocytes, and with soft tissue (*arrow*) can be observed (hematoxylin-eosin stain)

**Fig. 3:**
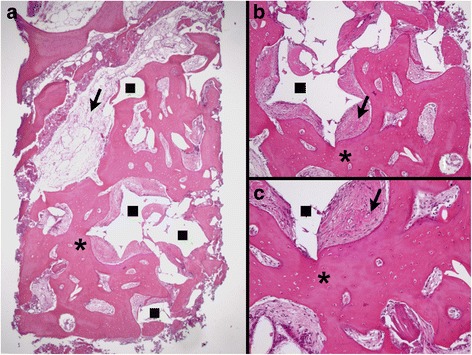
Histomicrograph of a biopsy from the BC + EMD group. Overview—×25 magnification; **b** ×30 magnification; **c** ×60 magnification. Areas corresponding to BC + EMD removed during histological processing (*square*) surrounded by newly formed bone (*asterisk*), with large numbers of osteocytes and soft tissue (*arrow*) can be observed. There is direct contact between the BC reminiscent, soft tissues, and vital bone (hematoxylin-eosin stain)

**Fig. 4:**
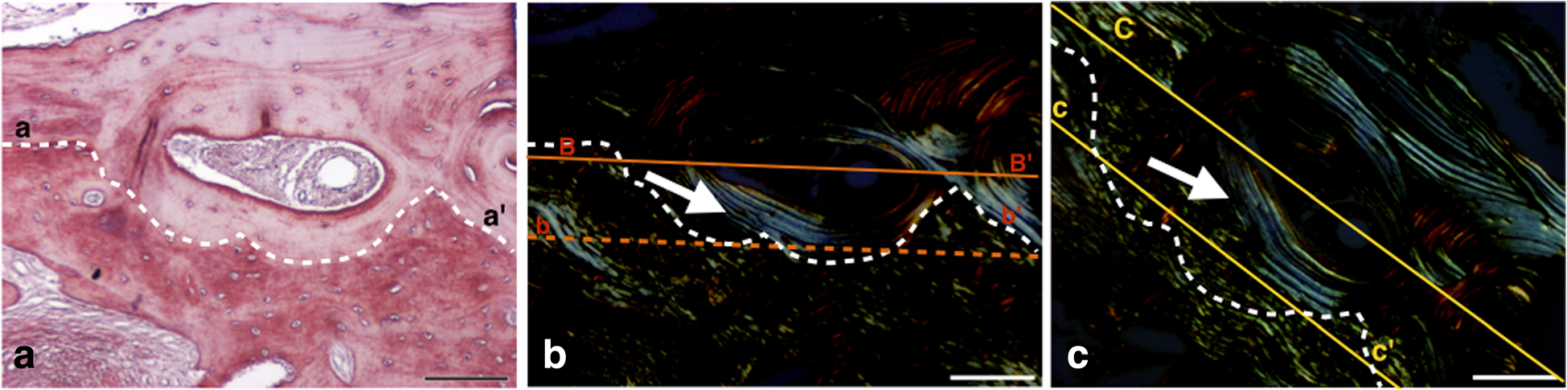
Histomicrograph of a biopsy from the BC group, showing an aspect of newly formed bone. Section stained with picrosirius-hematoxylin and digitalized with bright-field (**a**) and linearly polarized light (**b** and **c**). **b**, **c** Results of near 45° section rotation (between axes *B–B’* and *C–C’*) to compensate some of the orientation-related effects associated with linearly polarized light. In **a**, typical Haversian systems are showed (area observed above *dotted line*, *a* to *a’*). In **b** and **c**, the *arrows* indicate thin birefringent collagen bundle (appearing as *bright lines*) arranged around Haversian canals, suggestive of mature lamellar bone. The area observed below the *dotted line* is suggestive of immature (non-lamellar) bone, where collagen fibers undulations can be observed. The dark area corresponds to complete disorganization of the collagen fibers. *Bar* = 100 μm

**Fig. 5:**
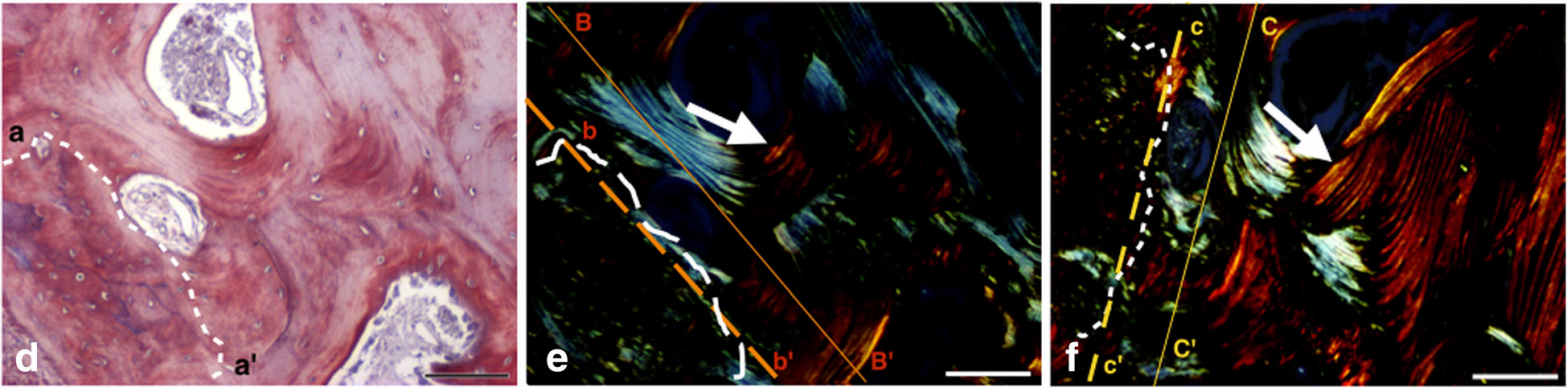
Histomicrograph of a biopsy from the BC + EMD group, showing an aspect of newly formed bone. Section stained with picrosirius-hematoxylin and digitalized with bright-field (**d**) and linearly polarized light (**e** and **f**). **e**, **f** Results of near 45° section rotation (between axes *B–B’* and *C–C’*) to compensate some of the orientation-related effects associated with linearly polarized light. In **d**, typical Haversian systems are showed (area observed above *dotted line*, *a* to *a’*). In **e** and **f**, the *arrows* indicate thin birefringents (appears visually as brilliance) collagen arrangement around Haversian canals suggestive of lamellar mature bone. Areas observed below *dotted lines* are suggestive of immature (non-lamellar) bone where collagen fiber undulations can be observed. The dark area corresponds to complete disorganization of the collagen fibers. *Bar* = 100 μm

The histological evaluation showed various amounts of newly formed bone, soft tissue, and remaining grafted material particles in all biopsies, with no apparent difference between groups (Figs. 2 and 3). In all samples, most part of the grafted material was removed due to decalcification during the histological processing. From the ghost images of the grafted material, the particles appeared in various forms and sizes, and in advanced stage of degradation. Evaluation under polarized light showed both areas of high birefringence in the newly formed bone, indicative of the high aggregation and organization of collagen bundles of mature lamellar bone, as well as areas of low birefringence, indicative of the disorganized collagen bundles of immature bone. No apparent differences in bone maturation were observed between the groups (Figs. 4 and 5). The new bone was in contact with the remaining graft particles at a variable extend within each biopsy, but again there were no apparent differences between the two groups. In all samples, only few inflammatory cells, mostly macrophages, were observed.

### Histomorphometric analysis

**Fig. 6:**
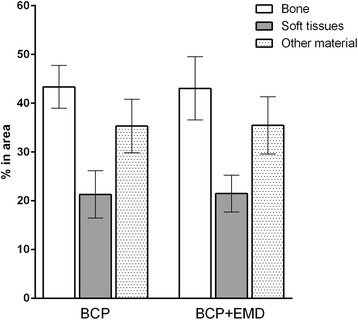
Histomorphometric evaluation results (considering six sections for each biopsy), for newly formed bone, soft tissues, and others

Within the ROI, mean bone area was 43.4% (SD 6.1; CI95 38.9–47.8) for the BC group and 43.0% (SD 9.0; CI95 36.6–49.5) for the BC + EMD group. The mean soft tissue area was 21.3% (SD 6.8; CI95 16.5–26.2) for BC group and 21.5% (SD 5.3; CI95 17.7–25.3) for BC + EMD group. The mean area of “other material” was 35.3% (SD 9.0; CI95 36.6–49.5) for BC group, and 35.5% (SD 8.2; CI95 29.6–41.3) for BC + EMD group. The data is graphically presented in Fig. 6. No differences between the groups were found for any of the three parameters evaluated (*p* value was 0.94 for bone, 0.96 for soft tissue, and 0.97 for other materials).

## Discussion

The present study compared the histological and histomorphometrical outcome of healing after maxillary sinus floor augmentation with BC with or without EMD, based on human biopsies. The results showed that addition of EMD did not enhance the outcome of healing, neither in terms of quality nor quantity of new bone. Nevertheless, the amount of bone generated after maxillary sinus floor augmentation with BC or BC + EMD was adequate to support successful implant placement and osseointegration of implants.

EMD is used for almost 20 years for enhancing tissue regeneration in periodontal defects, and it has been shown to exert anabolic action on several types of cells and factors relevant for bone regeneration [11, 12]. Nevertheless, there is still only sparse information from the clinic on the possible beneficial effect of adding EMD on a bone substitute material in terms of enhancing bone tissue regeneration in non-periodontal sites. In particular, a single study has previously evaluated the BC + EMD combination vs. BC in sinus lift, but due to the fact that only radiographic analysis was performed, the results were unclear [14]; thus, the present study, including histological evaluation, was performed. After 6 months of healing, about 43% of the evaluated part of the biopsy consisted of newly formed mineralized bone and about 35% consisted of grafting material; no differences between the groups were observed also in regard to bone tissue organization and maturation, as revealed by analysis of birefringence. Herein, only the 6 mm towards the apical aspect of the 10-mm-long biopsies was considered as the region of interest (ROI), in order to minimize any influence on the results from counting aspects of the alveolar ridge present before surgery.

Aiming to enhance bone formation and bone quality when bone substitute materials such as BC are used, biologics have often been added and positive results have occasionally been observed [16, 17]. The possibility that absence of any beneficial effect of EMD on bone regeneration herein was due to the sterile physiological saline solution added to the graft material mixture to facilitate its handling and transferring into the sinus, cannot be excluded. Indeed, the saline solution may have either diluted the concentration of EMD necessary to exert a beneficial effect or it may have interfered with adequate adsorption of EMD on the BC particles, resulting in altered (reduced) presence of EMD on the site during healing. In fact, in an in vitro study, published after the clinical procedures of the present study were concluded, it was shown that best adsorption of EMD on bone substitute particles is achieved when particles are dry and EMD is allowed to adsorb for at least 5 min. Further, in that study it was shown that inadequate adsorption of EMD on the bone substitute particles had negative influences in osteoblast proliferation and differentiation [14, 15, 18].

Nevertheless, the amount of bone generated with BC or BC + EMD herein was adequate to support successful implant placement and osseointegration of implants. In fact, more or less similar amounts of bone formation have been reported in studies evaluating human sinus biopsies after grafting with a variety of biomaterials (bone formation ranging approximately from 30 to 50%) [19]. On the other hand, an ideal situation would be that BC becomes gradually resorbed and completely substituted by vital bone tissue [8]. A few studies have indeed showed that biologics accelerate the degradation of biomaterials and consequently lead to larger bone formation at the grafted region [20, 21]. However, in the present study, EMD did not seem to influence graft remodeling in that manner. The possible biological and biomechanical long-term challenges of a loaded implant inserted in largely non-vital BC-grafted bone sites remain unknown. Recent studies, in fact, indicate high failure rates of implants inserted in sites augmented laterally and/or vertically with fresh-frozen allogeneic bone blocks [22], a material that remains largely necrotic for several months, despite good clinical graft incorporation [22–24]. In perspective, high long-term implant survival rates are reported after sinus augmentation with a variety of bone substitute materials [25].

## Conclusions

The present study showed that maxillary sinus floor augmentation with BC resulted in adequate amounts of new bone formation allowing successful implant installation, while adding EMD did not have a significant effect.
